# A seven-long noncoding RNA signature predicts overall survival for patients with early stage non-small cell lung cancer

**DOI:** 10.18632/aging.101550

**Published:** 2018-09-11

**Authors:** Ting Lin, Yunong Fu, Xing Zhang, Jingxian Gu, Xiaohua Ma, Runchen Miao, Xiaohong Xiang, Wenquan Niu, Kai Qu, Chang Liu, Qifei Wu

**Affiliations:** 1 Department of Hepatobiliary Surgery, The First Affiliated Hospital of Xi’an Jiaotong University, Xi’an 710061, Shaanxi, China; 2 Department of Surgical Intensive Care Units, The First Affiliated Hospital of Xi'an Jiaotong University, Xi’an 710061, Shaanxi, China; 3 Institute of Clinical Medical Sciences, China-Japan Friendship Hospital, Beijing 100029, China; 4 Department of Thoracic Surgery, The First Affiliated Hospital of Xi’an Jiaotong University, Xi’an 710061, Shaanxi, China; * Equal contribution

**Keywords:** long non-coding RNA, NSCLC, overall survival, risk score, stage I

## Abstract

Non-small cell lung cancer (NSCLC) is the most common cancer and cause of cancer-related mortality globally. Increasing evidence suggested that the long non-coding RNAs (lncRNAs) were involved in cancer-related death. To explore the possible prognostic lncRNA biomarkers for NSCLC patients, in the present study, we conducted a comprehensive lncRNA profiling analysis based on 1902 patients from Gene Expression Omnibus (GEO) and The Cancer Genome Atlas (TCGA) datasets. In the discovery phase, we employed 682 patients from the combination of four GEO datasets (GSE30219, GSE31546, GSE33745 and GSE50081) and conducted a seven-lncRNA formula to predict overall survival (OS). Next, we validated our risk-score formula in two independent datasets, TCGA (n=994) and GSE31210 (n=226). Stratified analysis revealed that the seven-lncRNA signature was significantly associated with OS in stage I patients from both discovery and validation groups (all *P<*0.001). Additionally, the prognostic value of the seven-lncRNA signature was also found to be favorable in patients carrying wild-type *KRAS* or *EGFR*. Bioinformatical analysis suggested that the seven-lncRNA signature affected patients’ prognosis by influencing cell cycle-related pathways. In summary, our findings revealed a seven-lncRNA signature that predicted OS of NSCLC patients, especially in those with early tumor stage and carrying wild-type *KRAS* or *EGFR*.

## Introduction

Non-small cell lung cancer (NSCLC) is one of the most common and lethal malignant diseases worldwide during the past decades due to lacking of early diagnostic and predictive biomarkers [1,2]. With the development of the molecular targeted agents [3] and the therapeutic strategy [4], the 5-year overall survival (OS) rate of NSCLC has been prolonged. However, only one third of NSCLC patients are diagnosed at an early stage [5]. To date, there are limited methods for us to provide a prognostic prediction of NSCLC patients, which is important to choose appropriate therapies for those patients [6]. Therefore, our study focused on finding a prognostic risk-score model derived from the whole-genome data.

Long non-coding RNAs (lncRNAs) are defined as non-protein coding transcripts longer than 200 nucleotides [7,8]. During the past decade, genome-wide sequencing helped researchers discover a large amount of lncRNAs [8]. Increasing studies suggested that the lncRNAs were involved in multiple cellular biological process and were associated with different human diseases including cancer [9,10]. Furthermore, several lncRNAs have been identified as oncogenes, such as *MALAT1* in lung cancer [11], *HOTAIR* in breast cancer [12], and *PANDA* in hepatocellular carcinoma [13]. Above lncRNAs were also found to be associated with the prognosis of cancer patients [13–15].

Recently, the prognostic values of lncRNAs have attracted much attention in the field of cancer research. Researchers put great effort into investigating the lncRNA biomarkers in multiple malignancies, including gastric cancer [16], breast cancer [17], hepatocellular carcinoma [18] and ovarian cancer [19]. In lung cancer, Tu *et al* explored the lncRNA biomarkers in based on 739 patients derived from GEO datasets [20] and identified an eight-lncRNA signature which was associated with clinical outcomes. In the present study, we conducted a large sample size (n=1902) and cross-platform (including RNA-seq and microarray data) analysis, and performed multiple validation. Considering the above clear advantages, our findings will provide robust evidence for the exploration of lncRNA biomarkers in NSCLC.

## RESULTS

### Identification of seven lncRNAs as prognostic biomarkers in discovery group

**Figure 1:**
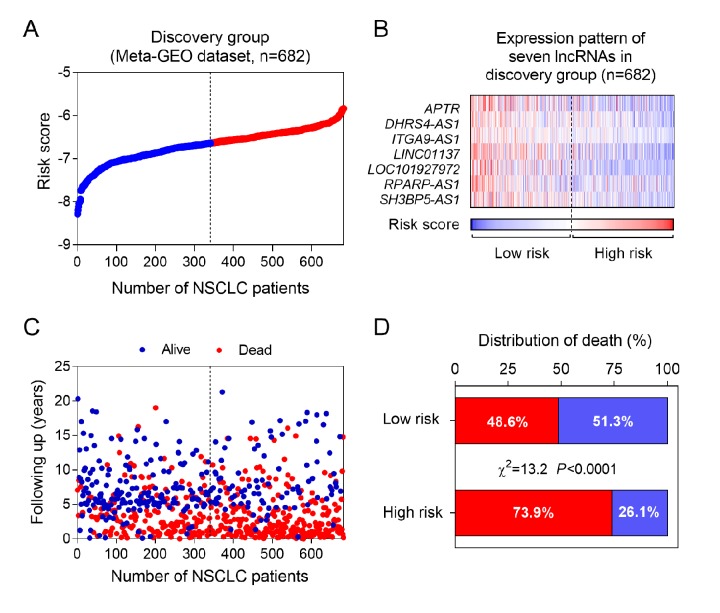
**The seven-lncRNA signature-based risk score in prognosis of overall survival in the discovery group.** (**A**) The seven lncRNA-based risk score distribution. (**B**) The heatmap of the seven lncRNA expression profiles. (**C**) The vital status of patients in high- and low-risk groups. (**D**) The mortality rate in low- and high-risk score groups.

By subjecting the lncRNA profiling data of discovery group to univariable Cox proportional hazards regression analysis, we obtained 457 lncRNAs whose *P*-value were less than 0.05. Among them, seven lncRNAs were selected for further analysis, including *APTR*, *DHRS4-AS1*, *ITGA9-AS1*, *LINC01137*, *LOC101927972*, *RPARP-AS1* and *SH3BP5-AS1*. Next, we fitted the 7 lncRNAs expression, the corresponding survival status and survival time into the multivariable Cox regression. Using the coefficients obtained from the multivariable Cox regression, a risk-score formula was constructed as following: risk score = - 0.06 x *APTR* - 0.20 x *DHRS4-AS1* - 0.35 x *ITGA9-AS1* - 0.23 x *LINC01137* - 0.25 x *LOC101927972* - 0.06 x *RPARP-AS1* - 0.20 x *SH3BP5-AS1*. Notably, in the formula, every lncRNA expression value is weight by a negative coefficient (Table 1). As shown in Figure 1A and 1B, the heatmap revealed that the expression level of seven lncRNAs was decreased accompanying with the higher risk scores. Furthermore, we calculated the association between risk score and cancer-related death (Figure 1C). Our data showed that mortality rate in high risk group was significant higher than low risk group (Figure 1D), indicating the seven lncRNAs might play a protective role in NSCLC.

**Table 1:** Seven lncRNAs significantly associated with the OS of NSCLC patients in the training group.

Gene name	Ensemble ID	Chr.	Coordinate	Z score	*P* value ^a^	Coefficient ^b^
*APTR*	ENSG00000214293	7	77,657,660-77,697,345	-3.25	1.16E-03	-0.06
*DHRS4-AS1*	ENSG00000215256	14	23,938,731-23,988,839	-3.14	1.69E-03	-0.20
*ITGA9-AS1*	ENSG00000235257	3	37,745,432-37,861,780	-2.83	4.62E-03	-0.35
*LINC01137*	ENSG00000233621	1	37,454,879-37,474,443	3.26	1.12E-03	-0.23
*LOC101927972*	ENSG00000269985	6	5,451,683-5,458,075	-2.98	2.89E-03	-0.25
*RPARP-AS1*	ENSG00000269609	10	102,449,817-102,461,106	-3.35	8.05E-04	-0.06
*SH3BP5-AS1*	ENSG00000224660	3	15,254,184-15,264,498	-2.92	3.45E-03	-0.20

**^a^** Derived from the univariable Cox proportional hazards regression analysis in the discovery group.

^b^ Derived from the multivariable Cox proportional hazards regression analysis in the discovery group.

### The prognostic values of seven-lncRNA signature in discovery and validation groups

**Figure 2:**
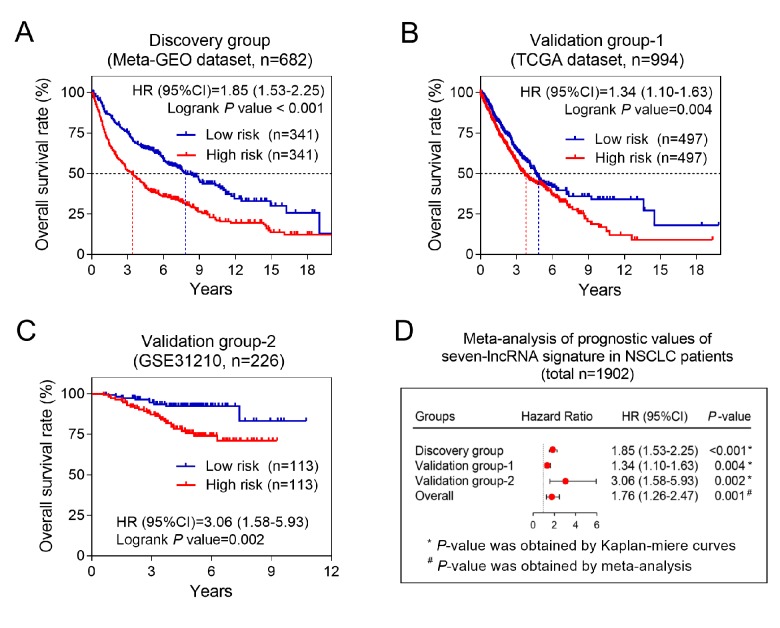
**The association between seven-lncRNA signature and overall survival in discovery and two validation groups.** Kaplan-Meier survival curves were plotted to estimate the overall survival probabilities for the low- versus high-risk group in the discovery group (**A**), validation group-1 (**B**) and validation group-2 (**C**). (**D**) Meta-analysis was performed using the prognostic results of three groups.

Considering the relationship between the seven-lncRNA signature and patients’ deaths, we next explored whether the seven-lncRNA signature could affect patients’ OS time. Our data in discovery group (Meta-GEO dataset, n=682) showed that the patients who had low risk-scores were supposed to have a longer OS time than higher risk group (Figure 2A). To validate above finding, we employed two validation groups, TCGA and GSE31210 dataset, containing 994 and 226 patients, respectively. As expected, patients in high risk group had a significant increased mortality risk than low risk group either in TCGA (HR=1.34, 95% CI= 1.10-1.63, *P* =0.004) or GSE31210 datasets (HR=3.06, 95% CI= 1.58-5.93, *P* =0.002) (Figure 2B and 2C). Moreover, prognostic meta-analysis based on above three groups (n=1902) confirmed that the seven-lncRNA signature was a risk factor for NSCLC patients (combined HR=1.76, 95% CI= 1.26-2.47, *P*=0.001) (Figure 2D).

### The seven-lncRNA signature was associated with OS in stage I patients

**Figure 3:**
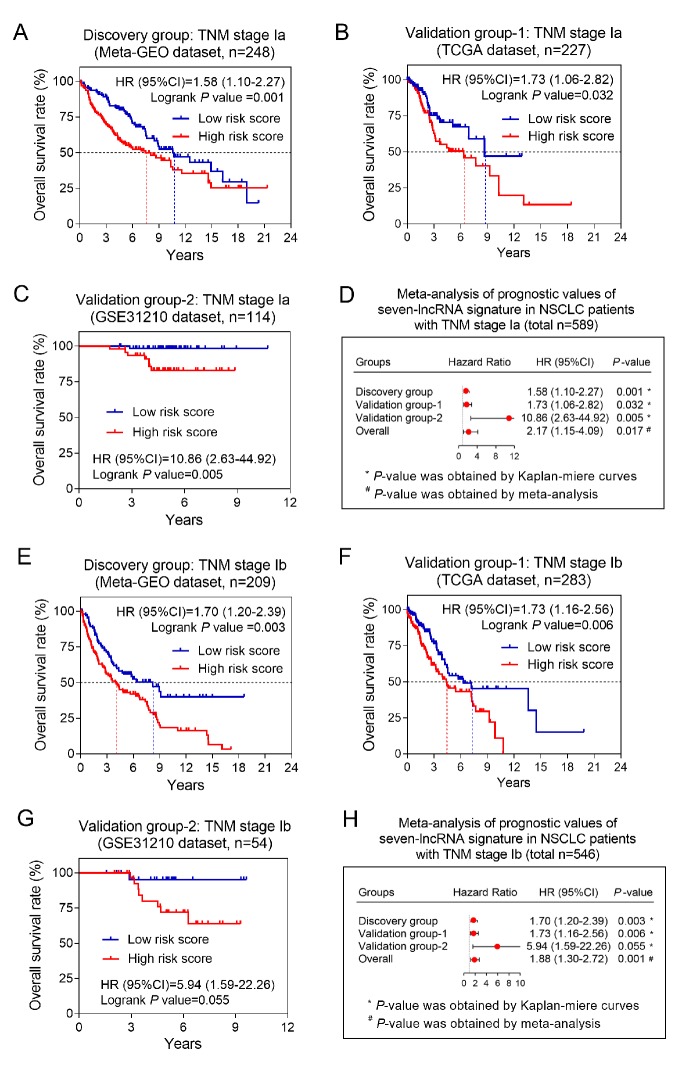
**The association between seven-lncRNA signature and overall survival in patients with stage I.** The Kaplan-Meier survival curves of discovery group (**A**), validation group-1 (**B**) and validation group-2 (**C**) were plotted, and meta-analysis (**D**) was conducted in patients with stage Ia. The similar results were obtained from the patients with stage Ib in discovery group (**E**), validation group-1 (**F**) and validation group-2 (**G**), and the prognostic meta-analysis (H) was also conducted.

To explore the impacts of clinical characteristics on the prognostic values of the seven-lncRNA signature, we performed a set of predefined stratified analysis. As shown in Table 2, we stratified the NSCLC patients by five clinical characteristics (including age, gender, smoking status, pathological subtypes and AJCC stage). According to the results of stratified analysis, the most significant result was found in stage I patients in both discovery and two validation groups (all *P*<0.001) (Table 2). To validate above findings, we further divided stage I patients into Ia and Ib according AJCC system, and calculated the prognostic values of the seven-lncRNA signature in each subgroups, respectively. Kaplan-Meier method was used to visualize the OS probabilities between high- and low-risk groups. As expected, the overall survival time of high risk group was all significantly shorter than low risk group in either stage Ia or Ib patients (Figure 3). The prognostic meta-analysis showed that the stage Ia patients with high risk scores had a two-fold increased mortality risk than those with low risk scores (combined HR=2.17, 95% CI= 1.15-4.09, *P*=0.017) (Figure 3D). Similarly, the association between the seven-lncRNA signature and OS also reached a statistical significance in stage Ib patients (combined HR=1.88, 95% CI= 1.30-2.72, *P*=0.001) (Figure 3H).

**Table 2:** The association between seven-lncRNA signature and OS of NSCLC patients in discovery and validating groups.

Variable	Discovery Group			Validation Group-1			Validation Group-2		
	Number (High/Low)	HR (95%CI)	*P* value	Number (High/Low)	HR (95%CI)	*P* value	Number (High/Low)	HR (95%CI)	*P* value
**Total**	**341/341**	**1.85 (1.53-2.25)**	**<0.001**	**497/497**	**1.34 (1.10-1.63)**	**0.004**	**113/113**	**3.06 (1.45-5.44)**	**0.002**
Age									
≤70	214/225	1.73 (1.36-2.16)	<0.001	291/284	1.30 (0.99-1.70)	0.056	111/110	3.18 (1.45-5.79)	0.003
>70	111/85	2.16 (1.61-3.28)	<0.001	189/206	1.39 (1.04-1.87)	0.029	2/3	1.24 (0.12-12.79)	0.855
Gender									
Male	203/252	1.72 (1.36-2.12)	<0.001	281/315	1.37 (1.06-1.75)	0.015	47/58	5.04 (1.52-9.20)	0.004
Female	138/89	1.95 (1.38-3.02)	<0.001	216/182	1.25 (0.91-1.75)	0.170	66/55	1.69 (0.64-4.52)	0.298
Smoking history									
Never smoker	23/5	2.31 (0.41-26.27)	0.275	57/32	2.07(1.10-5.46)	0.032	62/53	2.14(0.77-5.80)	0.152
Ever smoker	66/25	1.66 (0.80-3.83)	0.163	105/147	1.18(0.81-1.71)	0.398	51/60	3.84(1.33-7.68)	0.009
Current smoker	44/13	1.68 (0.75-4.54)	0.193	319/299	1.35(1.04-1.74)	0.023	0/0	NA	NA
Histology									
Squamous Carcinoma	55/136	1.32 (0.90-1.88)	0.170	185/309	1.41 (1.05-1.83)	0.020	NA	NA	NA
Adenocarcinoma	199/119	1.71 (1.30-2.44)	<0.001	312/188	1.26 (0.94-1.73)	0.120	NA	NA	NA
AJCC stage									
**Stage I**	**254/203**	**1.70 (1.35-2.23)**	**< 0.001**	**275/235**	**1.68 (1.25-2.31)**	**< 0.001**	**91/77**	**9.32 (2.27-15.35)**	**< 0.001**
Stage II	56/70	1.97 (1.24-3.02)	0.004	119/158	1.09 (0.75-1.57)	0.649	22/36	1.03 (0.40-2.66)	0.944
Stage III	24/59	1.13 (0.71-1.82)	0.608	74/89	1.05 (0.69-1.59)	0.818	0/0	NA	NA
Stage IV	6/7	3.09 (1.25-14.41)	0.030	21/11	1.25 (0.51-3.17)	0.607	0/0	NA	NA

Abbreviations: HR, Hazard ratio; 95%CI, 95% confidence interval; AJCC, the American Joint Committee on Cancer.

### The seven-lncRNA signature was associated with OS in patients carrying wild-type *KRAS* or *EGFR* genes

**Figure 4:**
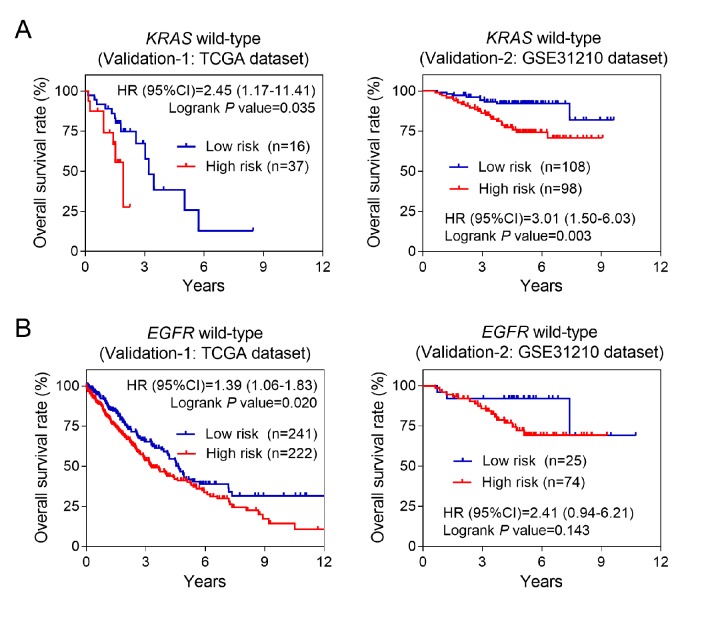
**Kaplan-Meier estimates of the overall survival of patients carrying wild-type *KRAS* or *EGFR* genes.** (**A**) Kaplan-Meier survival curves were plotted to estimate the overall survival for patients carrying wild-type *KRAS* gene in two validation groups. (**B**) Kaplan-Meier survival curves were plotted to estimate the overall survival for patients carrying wild-type *EGFR* gene in two validation groups.

*EGFR* and *KRAS* were the most frequent mutant genes and associated with poor prognosis in NSCLC. Previous studies suggested that the mutation frequency of above two genes was significantly decreased in patients with early stage. Bearing this in mind, we also performed stratified analysis based on *EGFR* or *KRAS* mutation status. Our data showed that the higher risk score was associated with higher mortality risk in wild-type *KRAS* or *EGFR* subgroups (Figure 4). Above results were consistent in the two validation groups, suggesting the seven-lncRNA signature acted as a risk factor for patients carrying wild-type *KRAS* or *EGFR* genes.

### Cell cycle-related pathways were associated with the seven-lncRNA signature

**Figure 5:**
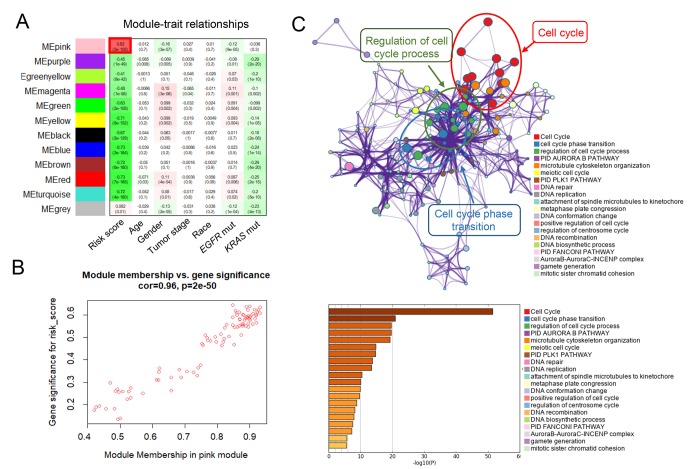
**WGCNA predicted biological pathways associated with the seven-lncRNA signature.** (**A**) The gene clusters obtained by WGCNA method. (**B**) The relationship between pink module membership and gene significance for risk score. (**C**) Significantly enriched pathways of the co-expressed genes in pink module.

Finally, to explore the potential mechanisms of the seven-lncRNA signature, we used the WGCNA method to cluster genes that highly correlated with the risk scores based on the profiling data of TCGA dataset [21]. We identified a total of 11 modules and found that pink module was the only positively correlated with the risk-score (*P* =2x10^-105^) (Figure 5A and 5B). Pathway enrichment analysis was then performed on an online-based web tool “Metascape” (http://metascape.org/) using the genes in pink module. As shown in Figure 5C, genes were significantly enriched in cell cycle-related pathways, including “cell cycle”, “cell cycle phase transition” and “regulation of cell cycle process” pathways, suggesting the activation of cell cycle-related pathways might contribute to higher mortality risk in patients with high risk scores.

## DISCUSSION

In the past decade, lncRNAs have been demonstrated by accumulating evidence to contribute in carcinogenesis and tumor progression [22]. In the present study, we conducted a seven-lncRNA signature, and validated their association with OS in two independent datasets. Moreover, we found the prognostic values of the seven-lncRNA signature reached a high statistical significance in patients with stage I and carrying wild-type *KRAS* and *EGFR* genes. To the best of our knowledge, this is the first study, which was based on the high-throughput lncRNA profiling data from nearly two thousand patients, to explore the lncRNAs as prognostic biomarkers for early stage NSCLC patients. Our findings might provide convincing evidence for clinical application.

In the present study, we identified a total seven lncRNAs which were associated with OS in NSCLC, including *APTR*, *DHRS4-AS1*, *ITGA9-AS1*, *LINC01137*, *LOC101927972*, *RPARP-AS1* and *SH3BP5-AS1*. Most of these identified lncRNAs were reported in cancer for the first time, except *APTR* (*Alu*-mediated p21 transcriptional regulator). Previous studies reported that *APTR* could promote the cell proliferation and contribute to carcinogenesis by repressing p21 promoter activities in human glioblastomas [21]. By contrast, in this study, we found that the coefficient of *APTR* was minus, indicating that *APTR* might play a protective role in NSCLC. The detailed molecular mechanism warrant further investigations.

The most important finding in this study was that the seven-lncRNA signature was significantly associated with OS in stage I patients. Our data showed their association reached a high statistical significance (*P*<0.001) either in discovery group or in validation groups, indicating the seven-lncRNA signature can provide a powerful prognostic tool for NSCLC with early stage. Recently, the oncogenic roles of *EGFR* and *KRAS* mutations in NSCLC have attracted increasing attentions. Patients carrying mutant *EGFR* or *KRAS* always had a poorer prognosis than those carrying wild-type genes [23,24]. Thus, the frequencies of wild-type *EGFR* and *KRAS* were always high in patients with early stage. We reviewed the *EGFR* and *KRAS* mutation status in TCGA-NSCLC dataset, and found that the mutation frequencies of *EGFR* and *KRAS* in stage 1 patients were only 10.2% and 5.7%, respectively, which were lower than advanced stage according to literature reports [25,26]. Interestingly, our data also showed that the seven-lncRNA signature was significantly associated with OS in patients carrying wild-type *EGFR* or *KRAS* genes, which might partially explain the favorable prognostic values of the seven-lncRNA signature in early stage patients.

In order to investigate the potential mechanisms affected by the seven lncRNAs, we also performed bioinformatic analysis. By using the WGCNA method, we clustered 11 gene modules from more than 5000 differentially expressed genes, and found that only one of them (pink) was positively correlated with seven-lncRNA signature. Pathway enrichment analysis further suggested that the genes in pink module were mostly enriched in cell cycle-related signaling pathways, indicating the seven-lncRNA signature might affect cell cycle-related pathways and consequently contributed to tumor progression.

In summary, we constructed a risk-score model derived from seven lncRNAs to predict the OS time of NSCLC patients. The risk association of the seven-lncRNA signature with survival was more evident in patients with early stage and carrying wild-type *EGFR* or *KRAS* genes. We warrant further studies, especially the large, well-performed cohorts to confirm or refuse our findings.

## MATERIALS AND METHODS

### Sample sources and study design

The raw data of gene expression and corresponding clinical information of NSCLC patients were downloaded from the Gene Expression Omnibus (GEO) and The Cancer Genome Atlas (TCGA) websites. Four GEO datasets (GSE30219, GSE31546, GSE37745 and GSE50081) were combined and used as the discovery group. After removal the five samples without survival data or enough clinical information, 682 patients from four GEO datasets were finally used for further analysis. Meanwhile, 994 patients from TCGA dataset were employed as validation group. Moreover, we also employed 226 patients from another independent GEO dataset (GSE31210) as the second validation group. All patients in GSE31210 belonged to the stage I and II of the American Joint Committee on Cancer (AJCC) system. The RNA expression profiling data of five GEO datasets were all performed on Affymetrix U133 Plus 2.0 microarray platform and the TCGA data was performed on Illumina sequencing platform. The clinical characteristics of discovery and validation groups were shown in Table 3.

**Table 3:** Clinical features of NSCLC patients in the training and validating groups.

Features	Discovery group (n=682)	Validation group-1 (n=994)	Validation group-2 (n=226)
Age (years), no (%)			
≤70	469 (71.7)	575 (58.7)	221 (97.8)
>70	196 (28.3)	404 (41.3)	5 (2.2)
Gender, no (%)			
Male	227 (33.3)	596 (60.0)	105 (46.5)
Female	455 (66.7)	398 (40.0)	121 (53.5)
Smoking status, no (%)			
Never smoker	28 (15.9)	89 (9.3)	115 (50.9)
Ever smoker Current smoker	91 (51.7) 57 (32.4)	252 (26.3) 618 (64.4)	111 (49.1) 0 (0)
Pathological grade, no (%)			
Squamous Carcinoma	191 (37.5)	494 (49.7)	NA
Adenocarcinoma	318 (62.5)	500 (50.3)	NA
AJCC stage, no (%)			
I	457 (67.3)	510 (51.9)	168 (74.3)
II	126 (18.6)	277 (28.2)	58 (25.7)
III	83 (12.2)	163 (16.6)	0 (0)
IV	13 (1.9)	32 (3.3)	0 (0)
KRAS Mutant type Wild type EGFR Mutant type Wild type	NA NA NA NA	24 (31.2) 53 (68.8) 101 (17.9) 463 (82.1)	20 (8.8) 206 (91.2) 127 (56.2) 99 (43.8)
Death status (%)			
Yes	418 (61.3)	394 (39.6)	9 (8.0)
No	264 (38.7)	600 (60.4)	104 (92.0)
Recurrence status (%)			
Yes	222 (39.4)	221 (22.2)	64 (28.3)
No	341 (60.6)	773 (77.8)	162 (71.7)

Abbreviations: AJCC, the American Joint Committee on Cancer; KRAS, Kirsten rat sarcoma viral oncogene homolog; EGFR, epidermal growth factor receptor.

### Normalization and lncRNA annotation of GEO data and TCGA data

The raw data of five GEO datasets (GSE30219, GSE31210, GSE31546, GSE37745 and GSE50081) were downloaded as probe-level CEL files. Then, all raw data were quantile normalized using Robust Multi-array Average (RMA) method. With the lncRNA-specific probes presented on Affymetrix U133 Plus 2.0 downloaded from Affymetrix website (http://www.affymetrix.com) [15], we finally identified 2986 lncRNA transcripts with RefSeq transcript IDs. As for TCGA data, we downloaded the sequencing data with the type of fragments per kilobase of exon per million fragments (FPKM) from the TCGA website (https://tcga-data.nci.nih.gov/).

### Construction of risk-score formula

After normalization and annotation of GEO raw data, we subjected 2986 lncRNAs to univariable Cox regression proportional hazards regression analysis to select lncRNAs which were associated with OS of NSCLC patients. Those lncRNAs with a statistical significance in univariable Cox regression were then selected into multivariable Cox regression to obtain the coefficients. By linearly combining the expression value of selected lncRNAs weighted by their coefficients, a risk-score formula was constructed as following [20]:

$$\text{risk score }=\;\sum_{i=1}^{N}\left({Exp}_{i}*{Coe}_{i}\right)$$

In our formula, the N is the number of 7, the ${Exp}_{i}$ are the expression value of every 7 lncRNAs and the$\;{Coe}_{i}$ are their corresponding coefficients from the multivariable Cox regression analysis [27].

### Survival analysis

According to above formula, the risk scores of NSCLC patients were calculated, by which patients were divided into high- and low-risk group with the cutoff of the median. The Kaplan-Meier method was used to assess the difference in survival time of high- and low- risk NSCLC patients. Difference of survival times between the two groups was considered as significant according to the *P* value of two-sided log-rank test less than 0.05.

### Prognostic meta-analysis

To investigate the combined prognostic values of the seven-lncRNA signature of discovery and two validation groups, we performed a prognostic meta-analysis. All meta-analysis was performed on STATA software, version 12.0 (StataCorp LP, College Station, TX, United States). Pooled HR value was calculated using the random-effects model.

### Weighted Correlation Network analysis (WGCNA)

To find the gene modules associated with our risk scores, we construct a co-expression network using the R package “WGCNA” according to our previous reports [28]. The soft thresholding power was selected to 9 to produce a weighted network. The enrolled genes were hierarchically clustered into 11 modules except the gray module.

### Pathway enrichment analysis

The pink modules, the most significant modules being associated with risk score, were picked out to perform the pathway enrichment analysis. Pathway enrichment analysis of genes in pink module was performed on an online-based web tool “Metascape” (http://metascape.org/).
